# Solitary fibrous tumor with IGF-II-induced non-islet cell tumor hypoglycemia: a case report and molecular characterization by next-generation sequencing

**DOI:** 10.3389/fonc.2023.1188579

**Published:** 2023-07-04

**Authors:** Helvijs Niedra, Ilze Konrade, Raitis Peculis, Sergejs Isajevs, Rihards Saksis, Roberts Skapars, Armands Sivins, Beate Elizabete Daukste, Dace Mezaka, Vita Rovite

**Affiliations:** ^1^ Department of Molecular and Functional Genomics, Latvian Biomedical Research and Study Centre, Riga, Latvia; ^2^ RigaEast Clinical University Hospital, Riga, Latvia; ^3^ Department of Internal Diseases, Riga Stradins University, Riga, Latvia

**Keywords:** non-islet cell tumor hypoglycemia, IGF-II, solitary fibrous tumor, whole genome sequencing, RNA sequencing

## Abstract

**Background:**

Non-islet cell tumor-induced hypoglycemia (NICTH) is a rare, life-threatening medical condition caused by excessive insulin-like growth factor II (IGF-II) secretion from tumors of most commonly mesenchymal origin. Using next-generation sequencing, we have characterized the genome and transcriptome of the resected IGF-II-secreting solitary fibrous tumor from a patient with severe hypoglycemia accompanied by hypoglycemia unawareness.

**Case presentation:**

A 69-year-old male patient presenting with abdominal discomfort was examined using computer tomography, revealing a large lesion at the lesser pelvis extending above the umbilicus. As no bone and lymph node metastases were detected, the patient was scheduled for laparotomy. Before surgery, the patient presented with symptoms of severe hypoglycemia. Suppressed C-peptide levels and subsequent hypokalemia indicated a possible case of NICTH. The patient was treated with methylprednisolone (8 mg) to assess hypoglycemia. After the surgery, mild hypoglycemia was present for the postoperative period, and no radiological recurrences were observed 3 and 12 months after discharge. Histopathological examination results were consistent with the diagnosis of malignant solitary fibrous tumor (SFT). Overexpression of IGF-II was confirmed by both immunohistochemistry and RNA sequencing. Further NGS analysis revealed an SFT characteristic alteration—*NAB2-STAT6* fusion. Additionally, three deleterious missense variants were detected in oncogenes BIRC6, KIT, and POLQ, and one homozygous in-frame deletion in the RBM10 tumor suppressor gene.

**Conclusion:**

While the *NAB2-STAT6* fusions are well characterized, the mutational landscape of SFTs remains understudied. This study reports the importance of NGS to characterize SFTs as we detected four coding variants in genes (*BIRC6, KIT, POLQ*, and *RBM10*) associated with tumorigenesis that could potentially contribute to the overall pathogenesis of SFT.

## 1 Introduction

Non-islet cell tumor-induced hypoglycemia (NICTH) is a rare neoplastic syndrome with a life-threatening prognosis (1). It is pathogenetically linked to the hypersecretion of insulin-like growth factor II (IGF-II) from IGF-II-producing tumors, also known as IGF-II-omas—a group of rare tumors of epithelial and mesenchymal cell origin (2). As a result, IGF-II-omas can induce hypoglycemia that often presents with neurogenic and neuroglycopenic symptoms, and in combination with hypoglycemia, unawareness can be fatal (3). The metabolic pathways activated by IGF-II involve insulin receptor (IR) and IGF receptor-I (1, 4). The activation of IRs inhibits both hepatic gluconeogenesis and glycogenolysis and increases glucose uptake in muscle cells (5). In NICTH, hypoglycemic effects are predominantly mediated through unprocessed IGF-II, also known as big IGF-II (2, 6). Since it has been found that the secretion of mature IGF-II from the tumor is relatively low compared to big IGF-II, possibly due to the generally high upregulation of IGF-II expression exceeding the cell’s capacity for post-translational processing (2). 80%–90% of processed IGFs are found in conjunction with IGF binding proteins (IGFBPs), forming ternary complexes that cannot cross capillary membranes, limiting IGFs’ interactions with insulin receptors (7). However, big IGF-II has a higher fraction that stays unbound or is found in binary complexes. As a result, big IGF-II is more potent than processed IGF-II as it can easily cross the capillary membrane and act on insulin receptors (8). Regarding the IGF-II-omas, a subgroup of fibroblastic and myofibroblastic tumors known as solitary fibrous tumors (SFTs) are regarded as one of the most common causes of NICTH (1). SFTs are characterized by a prominent, branching, thin-walled, dilated (staghorn) vasculature (9) and are primarily driven by *NAB2-STAT6* fusion (10). SFTs have an incidence rate of up to one case per million people per year (11), and based on histopathological and clinical characteristics, they are subdivided into benign, intermediate (NOS), and malignant categories (12). Interestingly, the overexpression of IGF-II in tumor tissue can be observed in up to 80% of SFT cases (13). However, in less than 5% of cases, the ectopic secretion is high enough to induce NICTH, also known as “Doege-Potter syndrome” in SFTs (14). This study presents a case of an intra-abdominal solitary fibrous tumor in a 69-year-old male patient with severe hypoglycemia accompanied by hypoglycemia unawareness. We have examined the clinical aspects of an NICTH case accompanied by molecular aspects of the tumor uncovered by the whole genome and transcriptome sequencing.

## 2 Materials and methods

### 2.1 Next-generation sequencing and RT-qPCR

For both the tumor and adjacent normal tissue samples, the DNA and RNA extraction was performed simultaneously from two 10-μm-thick formalin-fixed paraffin-embedded (FFPE) sections using the AllPrep DNA/RNA mini kit (Qiagen, Germany). For genome sequencing, the additional DNA sample extracted from white blood cells (reference genome) was obtained via the Genome Database of the Latvian population (LGDB) (15). Both tumor and reference DNA was sonicated with Covaris S220 (Covaris, USA) to attain 300-bp fragments for library preparation using MGIEasy Universal DNA Library Prep Set (MGI, China). RNA samples were treated for ribosomal RNA removal using the MGIEasy rRNA Depletion Kit (MGI, China), and the purified RNA library was prepared for NGS using MGIEasy Directional Library Prep Set (MGI, China). RNA and DNA samples were sequenced on the DNBSEQ-G400 sequencing platform (MGI, China). The tumor DNA sequencing target was 900 million (2 × 150 bp) reads, while the reference DNA sequencing target was 450 million (2 × 150 bp) reads. For RNA sequencing, the target reads were 25 million (2 × 150 bp). To further validate the expression on mRNA level of several genes (*BIRC6*, *RBM10*, *POLQ*, and *KIT*) which contained damaging variants, additional RT-qPCR analyses were performed on the ViiA 7 Real-Time PCR System (ThermoFisher Scientific, USA) with the extracted RNA. Input RNA (50 ng) was used for the RT-qPCR reaction, which was set up using QuantiNova SYBR Green RT-PCR Kit (Qiagen, Germany) and QuantiNova LNA PCR Assay oligos (Qiagen, Germany) with the following assay IDs: ENST00000288135 (*KIT*), ENST00000421745 (*BIRC6*), ENST00000264233 (*POLQ*), and ENST00000496012 (*RBM10*).

### 2.2 Tissue processing, histology, and immunohistochemistry

FFPE tissue was cut into 3-μm-thick sections. Antigen retrieval was achieved by treatment in a domestic microwave for 30 min in EDTA buffer pH=9.0 for immunohistochemistry. Sections were incubated in 3.0% H_2_O_2_/PBS to quench endogenous peroxidase activity and then blocked with Protein Block (Agilent, USA). The slides were then incubated for 1 h at room temperature with IGF-II rabbit monoclonal antibody diluted 1:100 (MA-32485, clone JJ092-3; ThermoFisher Scientific), STAT-6 antibody (Abcam, rabbit monoclonal antibody, ab32520), and RBM10 polyclonal antibody diluted 1:500 (Invitrogen, clone PA-5-83253). EnVision kit (Agilent, USA) was used to visualize the bonding of primary antibodies. 3'3-Diaminobenzidine-tetrahydrochloride (DAB) was then applied as chromogen for 7 min. Sections were counterstained in hematoxylin for 2 min. For positive control, a tissue sample from the human pancreas was used. Negative control was performed by omitting the primary antibody.

### 2.3 Data analyses

Tumor and reference samples from whole genome sequencing were analyzed using Nextflow (v20.04.0) nf-core/sarek pipeline (v2.7.1) (16, 17). Somatic single nucleotide and insertion/deletion variant calling was performed using Strelka (v2.9.10) (18). The following variant call format files were annotated using Ensembl Variant Effect Predictor (Ensembl release 107) (19) with variant allele frequency in 1000 genomes population cutoff set at 1%. For RNA-seq, rRNA reads were removed using SortMeRNA (v 4.3.4) (20) and mapped using Salmon (v1.6.0) (21) against GENCODE (*v38*) *Homo sapiens*. Differential expression analysis was done using R (v 3.5.3) and DESeq2 package (v1.20.0) (22). To validate the defined tumor type from histopathology, which used previously known tumor biomarkers, gene fusion analysis on RNA data was performed using STAR-Fusion (v1.11.1) (23). Results from this analysis were validated *in silico* using FusionInspector (v2.8.0) (24) and annotated using FusionAnnotator (v0.2.0) with CTAT Human Fusion Lib as the reference database.

## 3 Results

### 3.1 Clinical presentation

**Figure 1:**
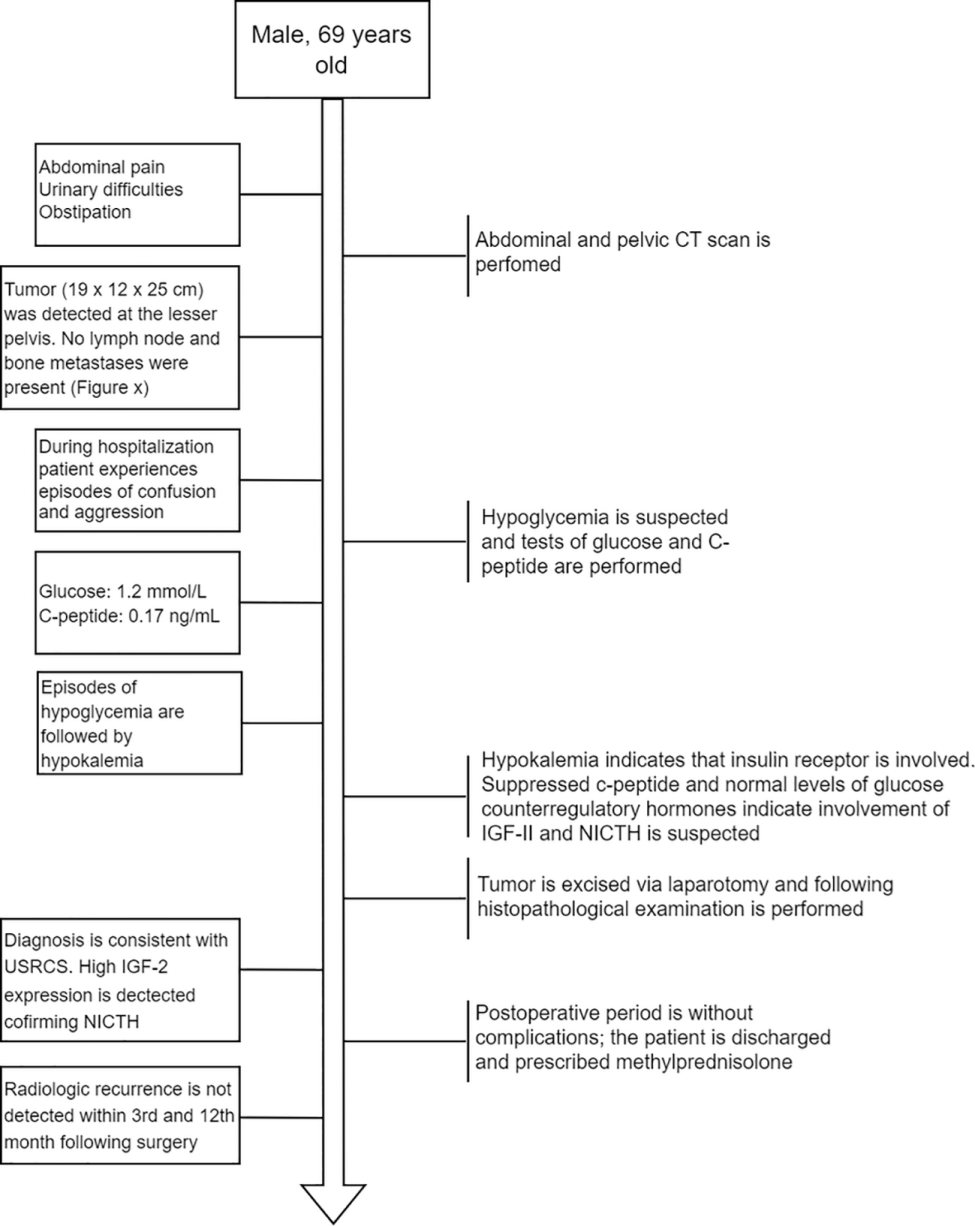
Flow diagram depicting the order of main events after the patient was admitted to the hospital.

**Figure 2:**
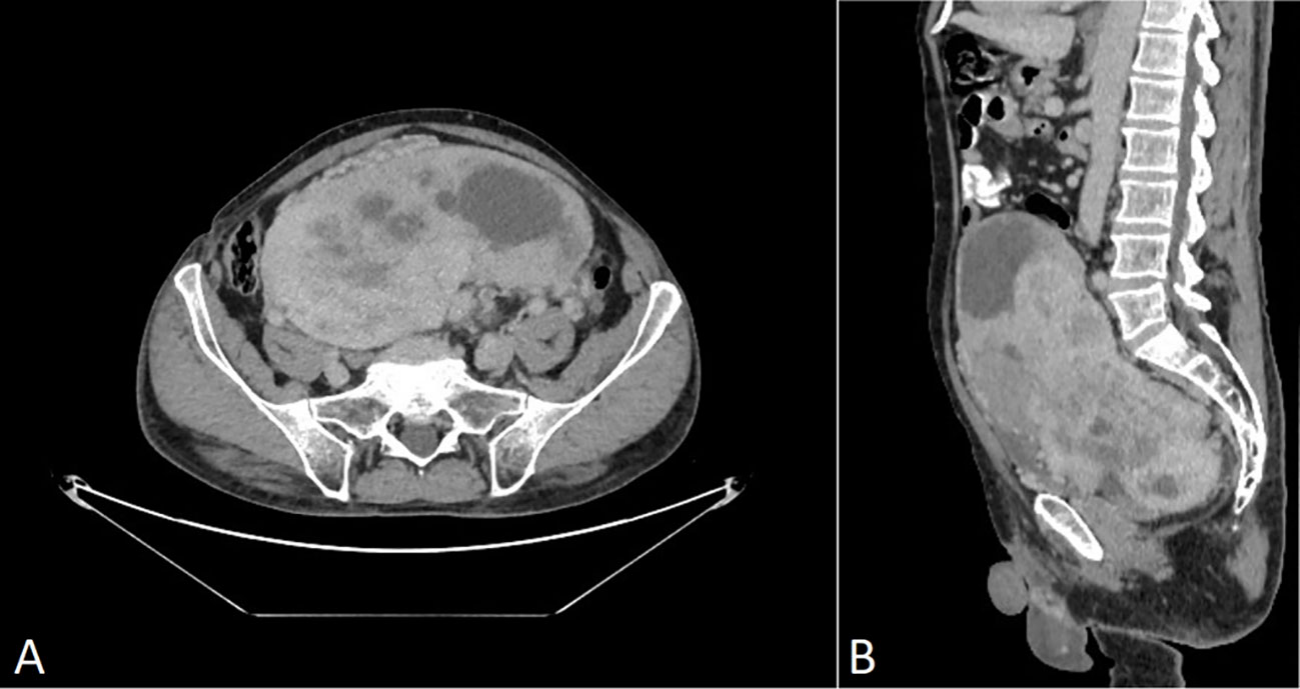
CT scan of venous post-contrast phase shows a large abnormal inhomogeneously vascularized smoothly contoured pelvic mass with avascular foci (necrosis). **(A)** Axial plane, **(B)** sagittal plane. No signs of perifocal infiltration in adjacent structures are present.

A 69-year-old man was admitted to the Riga East Clinical University Hospital with urinary difficulties and obstipation (**Figure 1**). For 10 years before being admitted, the patient suffered from discomfort in the abdominal area that prevented him from movements that required flexion of the back. Taking into account the patient’s complaints regarding the abdominal area, an abdominal and pelvic computed tomography (CT) scan was performed. The CT revealed an enormous pathological structure located in the lesser pelvis that extended slightly craniocaudally above the umbilicus (**Figure 2**). The size of the tumor was noted to be 19 cm **×** 12 cm **×** 25 cm (LL **×** AP **×** CC). Pathological lymph nodes and bone metastases were not detected. As a result, it was decided that a laparotomic tumor excision would be chosen as the treatment route.

While hospitalized, the patient experienced episodes of confusion and aggression. A psychiatrist consulted the patient about the episodes of confusion and aggression, and the patient’s glucose levels were also assessed. At the time, hypoglycemia of 1.2 mmol/L (reference interval: 3.30–5.89 mmol/L) with suppressed levels of C-peptide—0.17 ng/ml (reference interval: 0.9–4.0 ng/ml) was discovered. After the hypoglycemia episode, the patient suffered from hypokalemia, altogether indicating that the insulin receptor was involved (25). Following this, additional measurements of glucose counterregulatory hormones (cortisol and thyroid hormones) and IGF-I were taken. While the counterregulatory hormones were all within the reference range (**Supplementary Table 1**), a decreased level of IGF-I was detected (<15 ng/ml; reference interval: 43.0–220 ng/ml). Since elevated IGF-II levels are associated with suppressed plasma levels of insulin (C-peptide) and IGF-I (2, 26), IGF-II-mediated hypoglycemia was suspected. To avoid another episode of hypoglycemia before the surgery, the patient received methylprednisolone (8 mg).

The tumor was removed via open laparotomy, and it was removed in two portions as it was fixated within the minor pelvis. Urinary bladder and middle rectal wall marginal resections were performed. The internal iliac artery was ligated as it was infiltrated by a tumor. Overall blood loss was 2,700 ml. The patient received intraoperative and postoperative hemotransfusion. The postoperative period went without surgical complications. Only a mild hypoglycemia (3 mmol/l) episode was detected in the postoperative period. Ten days later, the patient was discharged from the hospital with instructions to continue methylprednisolone (8 mg the first 5 days, then 4 mg the following 2 days). The decision was made in correspondence with the relatively long half-life of IGF-II (10–16 h) (27). A follow-up abdominal and chest CT scan after the 3rd and 12th months revealed no radiological recurrences of the tumor.

### 3.2 Histopathological and immunohistochemical examination

**Figure 3:**
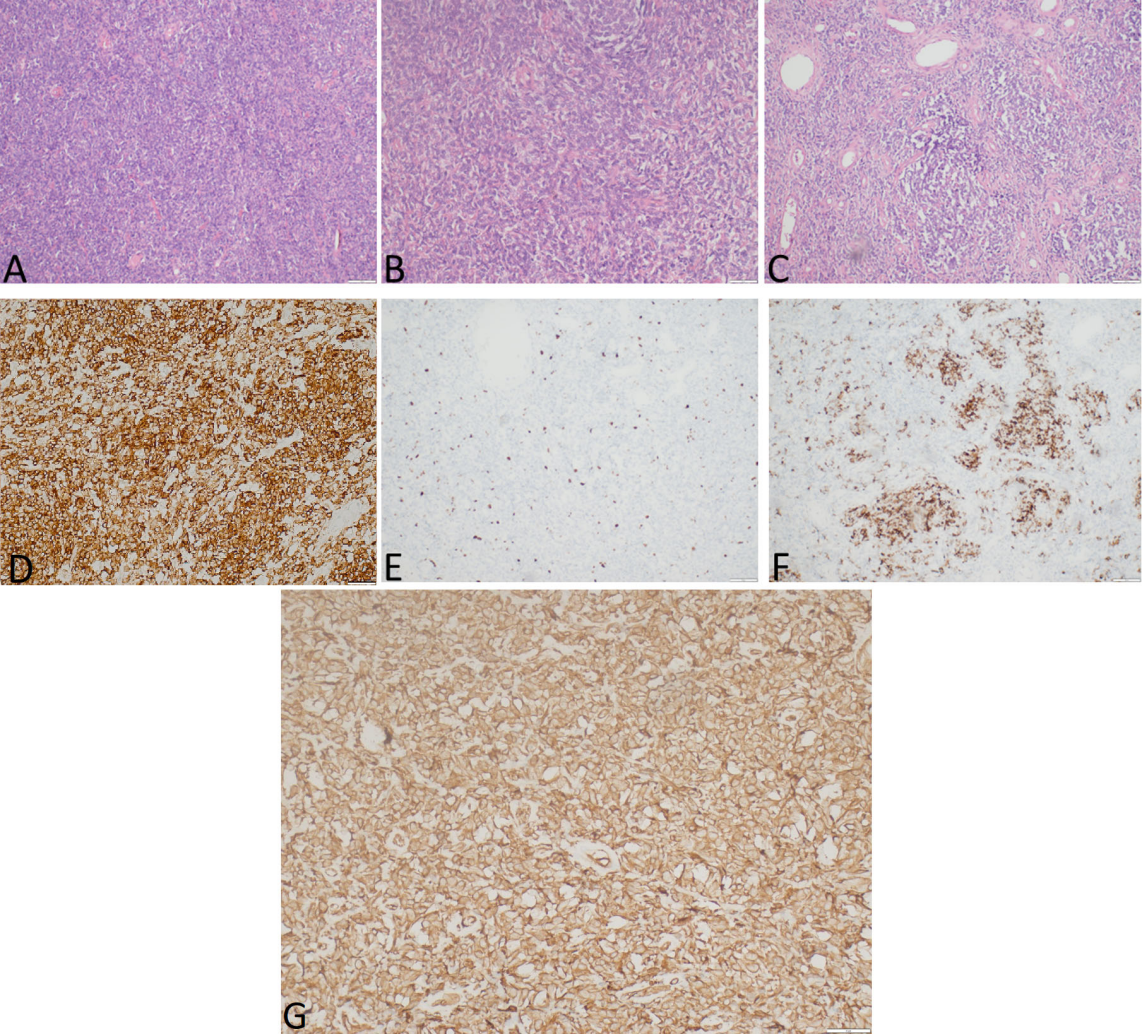
The representative hematoxylin-eosin **(A–C)** photomicrographs of a malignant solitary fibrous tumor: **(A)** Magnification ×40; **(B)** magnification ×200; **(C)** magnification ×100. **(D)** STAT6 expression, immunohistochemical staining method, magnification ×200. **(E)** Ki-67 expression, immunohistochemical staining method, magnification ×40. **(F)** Desmin expression, immunohistochemical staining method, magnification ×100. **(G)** IGF-II expression, immunohistochemical staining method, magnification ×200.

Gross examination demonstrated a tumor measured at 19 cm **×** 12 cm **×** 25 cm, weighing 2,480.0 g, gray and yellow colored, with multiple cystic areas. Accordingly, a histopathological examination of the tumor was performed (**Figure 3**). The tumor was composed of small to medium-sized round and/or spindled cells with limited eosinophilic or clear cytoplasm, predominantly arranged in cords, small nests, trabeculae, and pseudo-acinar structures in a fibrohyaline stroma. The tumor was CD99, vimentin, STAT6, and bcl-2 positive. Focal CD56, desmin, and NSE positivity were also observed. Ki-67 index was 10%, and mitotic activity was eight mitoses/10 HPF. The tumor cells were EMA, LCA, actin, CD34, CKAE1/AE3, S-100, NSE, and SOX-10 negative. The diagnosis was consistent with a malignant solitary fibrous tumor. The expression of IGF-II was also assessed and showed a strong IGF-II overexpression in tumor cells (**Figure 3G**). According to Demicco’s scoring system (28), the tumor was consistent with a high-risk tumor (total score: 6).

### 3.3 Variants identified by next-generation sequencing

**Figure 4:**
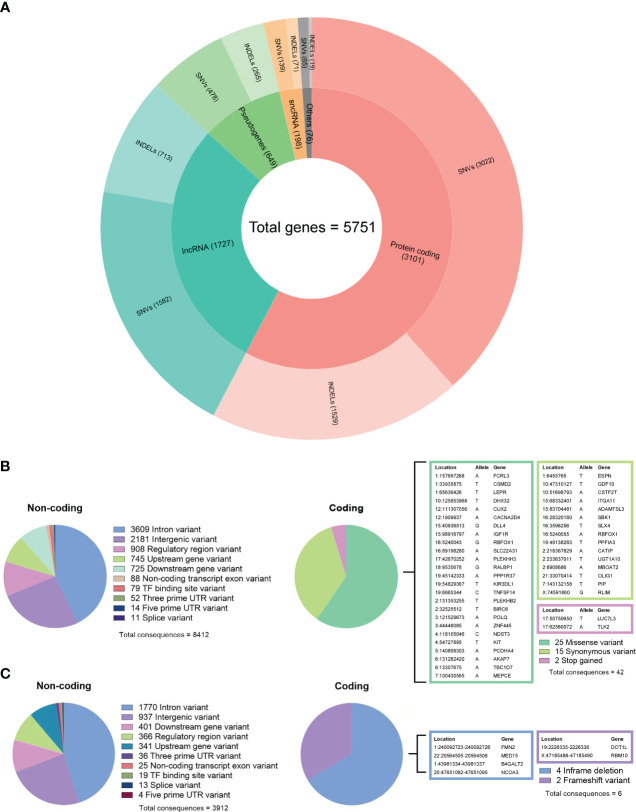
**(A)** Summary of gene biotypes containing 5,286 somatic single-nucleotide variants and 2,597 insertions/deletions; the inner layer represents the mutated gene count for each biotype, and the outer layer represents the variant count. **(B)** Summary of calculated non-coding and coding consequences caused by 6,589 single-nucleotide variants. **(C)** Summary of non-coding and coding consequences caused by 3,086 insertions and deletions; variants with coding consequences are listed in color-coded tables.

By comparing the tumor DNA with the white blood cell DNA, a total of 6,589 somatic single-nucleotide variants (SNVs) and 3,086 insertions and deletions (INDELs) were detected within the tumor DNA. Of these, 5,286 SNVs and 2,597 INDELs were located in 5,751 proteins, non-coding RNA, and pseudogenes (**Figure 4A**), while the remaining 1,303 SNVs and 489 INDELs were located in intergenic and regulatory regions. For both types of variants, the most affected genes were protein-coding and long non-coding RNA (lncRNA). Further analyzing the molecular consequences of all 6,589 SNV variants, we identified 8,412 non-coding consequences and 42 coding consequences (25 missense, 15 synonymous, and two stop gains) (**Figure 4B**). As for the INDELs, 3,912 non-coding and six coding consequences (four in-frame and two frameshift deletions) were identified (**Figure 4C**). A full list of variants annotated by Ensembl VEP can be found in **Supplementary Table 2**.

By further examining the variants using the COSMIC (v96 release), we identified 98 INDELs and 231 SNVs, which were spread across 194 cancer-associated genes listed in COSMIC Cancer Gene Census (CGC) (29). Of these variants, 38 SNVs and INDELs had been previously reported in other cancers as they had available COSMIC Genomic Identifiers (COSV) (**Supplementary Table 3**). Within these genes, most of the variants were identified as non-coding. As for the coding variants, three missense variants and one frameshift variant were identified (**Table 1**). According to both SIFT and Polyphen prediction algorithms, two variants, *BIRC6* (2:32525512-32525512) and *KIT* (4:54727895-54727895), were deleterious/probably damaging. The variant (3:121529673-121529673) in the *POLQ* gene was deleterious according to SIFT but benign according to Polyphen. Interestingly, while no predictions of deleteriousness were available for frameshift deletion variant 3:121529673-121529673 in the *RBM10* gene, it is worth mentioning that this variant was homozygous as it was localized in the X chromosome. To further validate the expression of these four mutated genes, we performed additional RT-qPCR analyses, which revealed that all four genes are stably expressed on the mRNA level (**Supplementary Figure 1A**). For the RBM10 gene, we carried out additional IHC analysis. Since the frameshift variant (X:47185486-47185490) in this gene results in a highly truncated form of the RBM10 protein, we expected the positivity of RBM10 to be relatively low within the IHC test. As expected, the RBM10 was only focally positive with nuclear expression in up to 10% of tumor cells (**Supplementary Figure 1B**), despite having a stable expression on the mRNA level.

**Table 1:** List of coding variants in COSMIC (v96 release) ccancer gene census genes.

Gene	Role in cancer	Variant pos	Ref/Alt	Normal locus coverage and VAF	Tumor locus coverage and VAF	Consequence	SIFT	Polyphen	CADD Phred	Existing variation ID
*BIRC6*	Oncogene	2:32525512-32525512	C/T	38/0 (0%)	43/50 (46%)	Missense variant	Deleterious (0)	Probably damaging (1)	26.8	COSV70204157
*KIT*	Oncogene	4:54727895-54727895	C/T	45/0 (0%)	50/49 (50%)	Missense variant	Deleterious (0.03)	Probably damaging (0.921)	23.6	rs891140054, COSV55398615
*POLQ*	Oncogene, TSG	3:121529673-121529673	C/A	41/0 (0%)	39/49 (44%)	Missense variant	Deleterious (0.01)	Benign (0.172)	18.15	–
*RBM10*	TSG	X:47185486-47185490	CAGTG/C	24/0 (0%)	36/0 (100%)	Frameshift variant	–	–	–	–

*VAF, variant allele frequency; Ref, reference allele; Alt, alternate allele; TSG, tumor suppressor gene; SIFT, Sorting Intolerant From Tolerant algorithm, which predicts whether an amino acid substitution affects protein function; Polyphen, predicts the possible impact of amino acid substation on the structure and function of the protein; CADD Phred, scores the deleteriousness of variants.

### 3.4 Next-generation sequencing of tumor RNA

The transcriptome sequencing was performed for both the tumor and adjacent normal tissue. Following this, we performed single sample differential expression analysis by comparing the tumor tissue against adjacent normal tissue. A total of 22 differentially expressed genes were identified (18 protein coding, three long non-coding RNAs, and one pseudogene), of which 12 were upregulated, and 10 were downregulated. Among these, a high upregulation was observed for the IGF-II gene (Log_2_FC = 7.9, *p*_adjusted_ = 0.02) and Lnc-INS-IGF2-1 (Log_2_FC = 8.1, *p*_adjusted_ = 0.02), a long non-coding RNA that is located in IGF-II gene sequence. Full differential expression analysis output is available in **Supplementary Table 4**.

Following the differential expression analysis, we further investigated gene fusions within the RNA sequencing data. A total of four intrachromosomal and one interchromosomal fusion were found in protein-coding genes (**Table 2**). For all five fusions, the breakpoints were identified at known splice sites and exon boundaries. In NAB2, exon 4 was fused with exon 4 of STAT6, PCDHGB2 exon 1 with PCDHGA10 exon 2, ASTN2 exon 19 with SUSD1 exon 9, KIAA1958 exon 2 with TRA2B exon 6, and PPP2R2D exon 6 with JAKMIP3 exon 2. The NAB2-STAT6 fusion was with the highest supporting fusion read count (146 reads), while the remaining four fusions had a supporting read count below 15.

**Table 2:** List of fusions discovered by RNA sequencing.

Fusion	Left gene exon/intron	Right gene exon/intron	Left gene breakpoint	Right gene breakpoint	Reads supporting fusion	Reads supporting non-fused allele at the left breakpoint	Reads supporting non-fused allele at the right breakpoint
*NAB2-STAT6*	Exon 4	Exon 2	chr12:57092968 (+)	chr12:57108299 (−)	146	45	13
*PCDHGB2-PCDHGA10*	Exon 1	Exon 2	chr5:141362556 (+)	chr5:141494807 (+)	14	1	5
*PCDHGB2-PCDHGA10*	Exon 19	Exon 9	chr5:141362556 (+)	chr9:112102285 (+)	3	5	1
*KIAA1958-TRA2B*	Exon 2	Exon 6	chr9:112575251 (+)	chr3:185921187 (−)	3	5	9
*PPP2R2D-JAKMIP3*	Exon 8	Exon 2	chr10:131947791 (−)	chr10:132104672 (−)	2	0	0

*+/− signs indicate DNA strand direction: (+), forward strand; (−), reverse strand.

## 4 Discussion

In this case report, we present the clinical aspects, treatment, and subsequent NGS analysis of a non-metastatic, high-risk SFT associated with NICTH. Using the NGS approach, we have investigated changes in both DNA and RNA levels within the tumor tissue where alongside *NAB2-STAT6* fusion, we identified several non-synonymous variants within the DNA that could potentially contribute to the overall pathogenesis of the SFT.

SFT typically shows a strong and diffuse expression of CD34 and nuclear STAT6 (30). In addition, bcl-2 and CD99 positivity can also be observed (30, 31). SFTs demonstrating a high mitotic activity with increased cellularity, necrosis, and infiltrative growth have traditionally been termed malignant (30). The new risk stratification models more accurately predict tumor behavior and prognosis (30). For risk stratification of patients, up to four scoring systems have been proposed, with the system proposed by Demicco et al. being the most successful (30) at determining the malignant potential by incorporating the patient’s age, tumor size, and mitotic activity (28). Upon examination of clinical and histological data, our study demonstrated a malignant solitary fibrous tumor that corresponded to Demicco’s risk system as a high-risk tumor, even though no metastases were detected. Our case also demonstrated positivity for STAT6, CD99, and bcl-2 and negativity for CD34. A previous study showed that the loss of CD34 can be specifically observed in the high-grade component of dedifferentiated SFTs and dedifferentiated SFTs with divergent differentiation (31).

The genetic hallmark of SFT is a paracentric inversion involving chromosome 12q, resulting in the fusion of the NAB2 and STAT6 genes (32). In our RNA data, we found *NAB2* exon 4 fused with *STAT6* exon 2 (**Table 2**), which is the most frequently reported *NAB2-STAT6* fusion in SFTs (33, 34). Despite this, the landscape of somatic mutations remains to be understudied as there are very few cases and NGS analysis is employed (32, 35, 36). However, these studies have employed a whole-exome or gene panel-based approach. In a recent exome sequencing study by Park et al., the most frequent alternation was *TERT* promoter mutation, which was detected in 7 out of 73 SFT samples studied. Within the study, the *TERT* promoter mutation also showed a significant association with aggressiveness, which further backs the initial observations by Demicco et al. and Bahrami et al., which showed that *TERT* promoter mutations are associated with worse outcomes (37, 38). In the Park et al. study, additional focus was also given to tumor suppressor *TP53* and apoptosis-related gene *APAF1* mutations, which were mutated in metastatic tissue. Furthermore, within functional experiments, an association between *TP53/APAF1* expression and malignant properties SFTs was discovered (32). In our study using the whole genome approach, we identified a total of 6,589 SNVs and 3,086 insertions/deletions, of which 33 variants caused a change in amino acid sequence according to VEP analysis (**Figures 4B, C**). While we did not find any of the variants that were reported in the aforementioned (32, 35, 36) large-scale sequencing studies of SFT, it is worth noting that we did find two intron variants in the *TERT* gene (**Supplementary Table 2**). In addition, we also detected 38 variants in cancer-associated genes reported by COSMIC (29), of which four variants had an impact on the amino acid sequence (**Table 1**). Of these, two missense variants—2:32525512-32525512 (*BIRC6*) and 4:54727895-54727895 (*KIT*)—had been previously reported as mesothelioma (39) and large intestine carcinoma (40). The missense variant in *POLQ* (classified as deleterious by SIFT algorithm) was unique because it has not been previously reported in either COSMIC or dbSNP databases. *POLQ* encodes DNA polymerase θ, which is involved in processes regarding the repair of double-stranded breaks in DNA, chromosomal translocations, and base excision repair. Therefore, it is unsurprising that alterations of *POLQ* have been reported in multiple malignancies and are often associated with poorer outcomes (41). Lastly, we detected a homozygous deletion causing frameshift of the *RBM10* gene, which regulates the splicing of genes involved in NOTCH1 signaling (42). As this gene was located in the X chromosome of a male patient, the homozygous allele was expected. Mutations of this gene have been reported in lung adenocarcinomas (42, 43).

Overexpression of *FGFR1*, *ALDH1A1* (*ALDH1*), *EGFR*, *JAK2*, histone deacetylases, and retinoic acid receptors has been frequently observed in SFT (44). In our data, we found significant overexpression of *ALDH1* isoform *ALDH1A2* in tumor tissue, a gene that can be overexpressed in T-cell acute lymphoblastic leukemias (45). In addition, it has been demonstrated that SFT has the potential to produce big IGF2 and cause hypoglycemia. Although many SFTs exhibit overproduction of IGF2, in only less than 5% of cases can a symptomatic hypoglycemia be observed (13, 14). In our case, the patient presented with symptoms of confusion and aggression, which pointed to severe hypoglycemia. The pathognomonic feature of NICTH is the IGF-II:IGF-I ratio (5); however, this assay is unavailable in Latvia, and therefore, it could not be checked. Despite this, the laboratory findings of the patient were self-explanatory: suppressed C-peptide levels, normal range of counterregulatory hormones (cortisol and thyroid hormones), suppressed IGF-I levels, and hypokalemia. Therefore, insulinoma was ruled out as a differential diagnosis. As NICTH was suspected, methylprednisolone (8 mg) was administrated to counter the effects of hypoglycemia. Following the resection of the tumor, the overexpression of IGF-II was confirmed both histologically (**Figure 3G**) and by RNA-seq (**Table 3**).

**Table 3:** Results of differential expression analysis.

Gene	Log_2_FC	*p*_adjusted_	Function
*ACTG2*	−8.6	0.02	Smooth muscle contraction
*AHNAK2*	−8.3	0.02	May be involved in calcium signaling
*ALX4*	9.6	0.02	Transcription factor
*FLNC*	−8.1	0.02	May be involved in reorganizing the actin cytoskeleton in response to signaling events
*IGF2*	7.9	0.02	Possess growth-promoting activity
*MMP3*	8.5	0.02	Extracellular matrix degradation
*MYH11*	−8.9	0.02	Muscle contraction
*XIST*	−9.1	0.02	lncRNA
*Lnc-INS-IGF2-1*	8.1	0.02	lncRNA
*SYNM*	−7.8	0.03	Type-VI intermediate filament, which plays an important cytoskeletal role within the muscle cell cytoskeleton
*TNC*	−7.8	0.03	In tumors, stimulates angiogenesis by elongation, migration, and sprouting of endothelial cells
*CACNA1H*	−7.2	0.04	Subunit of voltage-sensitive calcium channels that are involved in muscle contraction, hormone or neurotransmitter release, gene expression, cell motility, cell division, and cell death
*CNN10*	−7.5	0.04	Thin filament-associated protein is implicated in the regulation and modulation of smooth muscle contraction
*COL17A1*	7.4	0.04	May play a role in the integrity of hemidesmosome and the attachment of basal keratinocytes to the underlying basement membrane
*GABRA2*	7.1	0.04	The major inhibitory neurotransmitter in the vertebrate brain
*GPR88*	7.4	0.04	May play a role in the regulation of cognitive and motor function
*JPH2*	−7.4	0.04	Provides a structural foundation for functional crosstalk between the cell surface and intracellular calcium release channels
*KERA*	7.1	0.04	May be important in developing and maintaining corneal transparency and for the structure of the stromal matrix
*PTPRVP*	7.3	0.04	Pseudogene
*CARMN*	−7.0	0.04	lncRNA
*ALDH1A2*	6.8	0.04	Recognizes as substrates free retinal and cellular retinol-binding protein-bound retinal
*MCOLN3*	6.9	0.04	A nonselective ligand-gated cation channel probably plays a role in the regulation of membrane trafficking events

*Log_2_FC, Log_2_-transformed fold change in gene expression; p_adjusted_, FDR-corrected p-value.

While the exact mechanisms of IGF-II overexpression in SFTs remain unexplored, the study by Hajdu et al. proposed that the overexpression of IGF-II may be linked to loss of imprinting (LOI) due to mutations in the *IGF2* gene (46). *ApaI* polymorphisms located in exon 9 (3' UTR region) of *IGF2* are often detected in SFTs presenting with high tissue IGF-II expression (46). However, in our case, we did not detect any polymorphisms within *ApaI* sites of the *IGF-2* gene. In our case, overexpression of IGF-II could be attributed to the *NAB2-STAT6* fusion as this change upregulates the expression of *EGR1*, which is the transcriptional activator of IGF-II (47–49). It has also been implied that IGF-II can serve as a therapeutic target for tumors harboring *NAB2-STAT6* mutations (49).

In clinical practice, it is important to account for the half-life of IGF-II, as the effects could still affect the patient even after the tumor resection (27). Approximately 75% of circulating IGFs are bound in 150 kDa ternary complexes consisting of either IGF-I or IGF-II and IGFBP-3 (IGF binding protein 3)/ALS (acid-labile subunit) (50, 51). Within circulation, these complexes increase the half-life of IGFs from 10 min to 10–16 h, serving as a long-lasting reservoir of IGF (50, 51). As a result, methylprednisolone treatment was continued for up to 7 days after discharge from the hospital.

Altogether, this study reports a rare case of NICTH causing solitary fibrous tumors investigated by whole genome and whole transcriptome sequencing. Here, we would like to propose the inclusion of NGS analysis in future cases as it would bring a better understanding of molecular mechanisms behind SFTs and facilitate the development of more advanced risk stratification systems. As in addition to the classical *NAB2-STAT6* fusion, we report four coding variants in cancer-associated genes (*BIRC6, KIT, POLQ*, and *RBM10*) which have not been previously reported in SFTs.

## Data availability statement

The datasets presented in this article are not readily available because of ethical and privacy restrictions. Requests to access the datasets should be directed to the corresponding author/s.

## Ethics statement

The studies involving human participants were reviewed and approved by The inclusion of human participants in this study was reviewed and approved by the Central Medical Ethics Committee of Latvia (protocol: Nr. 01-29.1.2/2659). The patients/participants provided their written informed consent to participate in this study. Written informed consent was obtained from the individual(s) for the publication of any potentially identifiable images or data included in this article.

## Author contributions

HN performed sample preparation for sequencing, sequencing data analyses, and manuscript preparation. IK was responsible for clinical data collection, case management, manuscript preparation, and revising (HN and IK contributed equally as the first authors). RP and RSa contributed to the additional sequencing data analyses and revisions. SI contributed to preparation of the histopathological report and manuscript. RSk, AS, BD, and DM contributed to clinical data collection. VR contributed to manuscript preparation and supervision of the study. All authors contributed to the article and approved the submitted version.
