# Supplementary figures and images for: Solitary fibrous tumor with IGF-II-induced non-islet cell tumor hypoglycemia: a case report and molecular characterization by next-generation sequencing
Source: Front Oncol. 2023 Jul 4;13:1188579. doi: 10.3389/fonc.2023.1188579 (PMC10352493; doi:10.3389/fonc.2023.1188579)

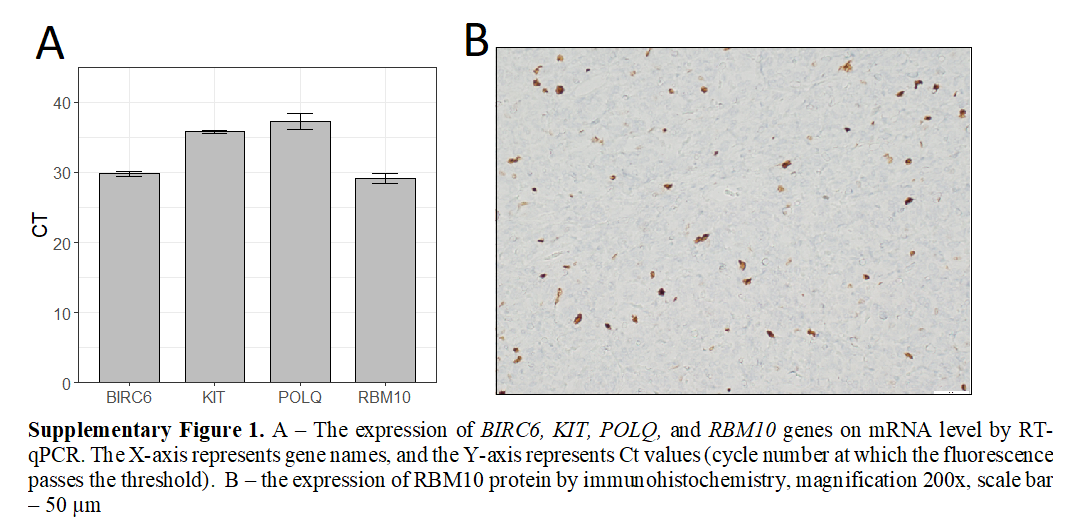

Supplement: Supplementary file 1 [file Image_1.tiff]
